# Relevance of inferior petrosal sinus sampling in the diagnosis of Cushing’s syndrome: a case report

**DOI:** 10.1515/almed-2022-0104

**Published:** 2022-11-28

**Authors:** Clara Jiménez García, Paula Sirera Sirera, María Eugenia Torregrosa Quesada, Victoria González Bueno, Rocío Alfayate Guerra

**Affiliations:** Clinical Laboratory, Hospital General Universitario de Alicante, Alicante, Spain; Hormone Testing Laboratory, Hospital General Universitario de Alicante, Alicante, Spain

**Keywords:** catheterization, corticotropin-releasing hormone (CRH), Cushing’s syndrome

## Abstract

**Objectives:**

Non-specific signs and symptoms of Cushing’s syndrome (CS) can pose a diagnostic challenge.

**Case presentation:**

We report the case of a man referred to the service of endocrinology for suspected CS. Hypercortisolism was confirmed on CS screening tests, whereas diagnostic tests confirmed the presence of adrenocorticotropin (ACTH) -dependent CS. The corticotropin-releasing hormone stimulation (CRH) test was performed to determine whether CS had an endogenous or ectopic origin. Since the CRH and the magnetic resonance imaging (MRI) test were negative, IPSS was performed and suggested that CS was originated in the pituitary glands. Transsphenoidal pituitary resection was carried out. Histopathological analysis confirmed the cortitotrope origin of the tumor.

**Conclusions:**

The etiological study and differential diagnosis of CS are complex processes that involve a variety of biochemical and imaging tests. It is important that a sequence of biochemical screening and diagnostic tests is performed, along with studies for establishing the location of the lesion, to determine whether CS has an adrenal, pituitary or ectopic origin. Despite its good diagnostic performance, the results of biochemical tests may not be conclusive, especially in ACTH-dependent CS. In the case reported, the inconclusive results obtained in the CRH test rendered an invasive procedure (IPSS) necessary, which ultimately confirmed diagnosis.

## Introduction

The diagnosis of Cushing’s syndrome (CS) (suprarrenal origin) is challenging, since it may be difficult to distinguish its signs and symptoms from those of other illnesses.

To ensure this rare condition does not remain unnoticed, it is necessary that a sequential diagnostic protocol is established. Firstly, the protocol should facilitate the identification of patients with endogenous hypercortisolism, based on clinical data and simple tests. Then, other more complex specific studies should be performed to determine the origin of hypercortisolism and establish a treatment [1, 2].

Since none of the diagnostic tests for CS have a sensitivity >90%, an invasive procedure, such as inferior petrosal venous sinus sampling (IPSS), may be necessary [3, 4].

Initial screening for CS should include two determinations of 24-h urine free cortisol (UFC), serum cortisol after nocturnal suppression with the administration of 1 mg of dexamethasone, and/or nocturnal salivary cortisol (NSC) at 23 h [5]. Two positive UFC results confirm diagnosis.

Etiologic diagnosis is based on the measurement of adrenocorticotropin (ACTH) concentrations [1] to determine whether or not CS is ACTH-dependent. Values of >15 pg/mL are suggestive of ACTH-dependent CS [5].

Differential diagnosis of pituitary vs. ectopic ACTH-dependent CS is based on the measurement of serum cortisol after suppression with 8 mg of dexamethasone (specificity 67%, sensitivity 79%), complemented with the corticotropin-releasing hormone stimulation (CRH) test (specificity 88–93%, sensitivity 91–100%). In the majority of pituitary tumors, corticosteroids at high doses suppress ACTH secretion, which does not occur with ectopic neuroendocrine tumors [6]. In the CRH test for adults, 100 µg of synthetic CRH are injected intravenously; then, timed blood samples are collected to measure cortisol and ACTH concentrations. An increase of >20% in plasma cortisol or >50% in ACTH with respect to the baseline value is suggestive of Cushing’s disease (CS).

Magnetic resonance imaging (MRI) of the pituitary gland may be useful to identify the location of the tumor, although results are not always conclusive.

When the results of biochemical tests are inconsistent or lesions are not found on MRI, an IPSS may be necessary. IPSS involves drawing samples from the venous drainage from the pituitary gland to the cavernous sinus and from the cavernous sinus to the inferior petrosal sinuses (IPS). Following the bilateral catheterization of the IPS, ACTH is measured at baseline and after CRH stimulation (at 3, 5, and 10 min) in samples from the inferior petrosal sinuses and in peripheral blood samples, collected simultaneously. A central/peripheral ACTH (C/P) gradient of ≥2 in basal samples or ≥3 in post-stimulation samples are suggestive of CD. A right/left (or vice versa) gradient of ≥1.4 between the two IPS suggests that the tumor is located in the side of the petrosal sinus with the highest ACTH concentration [1, 2].

In this report, we demonstrate the extent to which this condition may be challenging.

## Case presentation

We report the case of a 34 year-old man referred from the Service of Endocrinology on suspicion of CS. Suspicion was based on the presence of osteopenia/osteoporosis on bone density test, which was performed for stress metatarsal microfractures, weight gain, red face, and irritability. The patient did not have any personal or familial history of interest. Physical examination revealed a round face, mild HTN, and a fat lump between the shoulders. Abdominal reddish stretch marks were not found.

Laboratory results, including liver and kidney function tests, where within normal range. After exogenous intake of steroids was excluded, and upon suspicion of endogeous hypercortisolism, screening tests for CS were performed. Serum, salivary and urine cortisol (after extraction with dichloromethane), and ACTH concentrations were measured by electrochemiluminescence (ECLIA) on a Cobas analyzer (Roche Diagnostics). Results were as follows: UFC1 818 μg/24 h and UFC2 636.5 μg/24 h (VR: 10–120), suppression with 1 mg of dexamethasone 14.3 μg/dL (VR<1.8) and NCS 1.22 μg/dL (VR<0.27). These results confirmed an endogenous origin of CS. The presence of elevated ACTH concentrations at baseline (71 pg/mL (VR: 9–52)) in relation to cortisol levels were suggestive of ACTH-dependent CS.

Of the tests performed to identify the origin of ACTH secretion, cortisol suppression with 8 mg of dexamethasone suggested a pituitary origin (from 21 μg/dL to 5.9 μg/dL, reduction of >50%). However, CRH test did not demonstrate an increase of >50% or >20% in ACTH or cortisol, which are diagnostic criteria for CD (Table 1) [5]. MRI of the pituitary gland ruled out the presence of adenoma.

**Table 1:** CRH^a^ test results.

Time, minutes	Cortisol, μL/dL	Cortisol increase (>20%)	ACTH, pg/mL	ACTH increase (>50%)
Baseline	14.7	–	46	–
15′	16.4	11.56	46.2	0.44
30′	13.2	−10.20	39.7	−13.7
45′	11.6	−21.09	38.9	−15.43
60′	10.7	−27.21	34.3	−25.43
90′	10.1	−31.29	33.8	−26.52
120′	8.9	−39.46	38	−17.39

^a^Endovenous administration of 1 μg/kg of corticotropin-releasing hormone and timed sampling of blood to measure cortisol and ACTH (adrenocorticotropin) concentrations.

Given the inconsistency of results, IPSS was performed after informed consent was obtained from the patient. The ACTH (C/P) gradient following CRH stimulation was >3, which confirmed a pituitary origin (Table 2). The gradient suggested a right-sided location of the tumor.

**Table 2:** IPSS^a^ results.

Time, minutes	ACTH peripheral, pg/mL	ACTH right, pg/mL	ACTH left, pg/mL	Right-side/peripheral gradient	Left-side/peripheral gradient	Inter-sinus LIPS/RIPS	Inter-sinus RIPS/LIPS
**0′**	29.93	35.31	37.59	1.17	1.25	1.06	0.93
**3′**	30.92	111.6	39.75	**3.60**	1.28	0.35	2.80
**5′**	34.13	189.9	181.1	**5.56**	**5.30**	0.95	1.04
**10′**	38.12	309.6	147.7	**8.12**	**3.87**	0.47	2.09

^a^Endovenous administration of 100 µg of corticotropin-releasing hormone and sampling of blood from the inferior petrosal sinuses (right and left) and from peripheral blood to measure ACTH concentrations at 0, 3, 5 and 10 min. Values above the ratio are given in bold.

Transsphenoidal resection surgery was performed. In the histopathological study, immunohistochemistry was negative for ACTH. However, the availability of the transcription factor Tpit test in our center helped us identify the corticotrophic origin of the tumor. Tpit specifically regulates the expression of the proopiomelanocortin (*POMC*) gene in corticotrophic tumors [3]. The inclusion of this test in our protocol has enabled our Service to correctly classify some subtypes of pituitary tumors that would otherwise have been classified as null cell.

## Discussion

Diagnosis of CS is simple when the clinical signs and symptoms and the results of biochemical and imaging studies are consistent, which make it possible to correctly identify the location of the tumor. However, as it occurred in the case reported, the diagnosis of ACTH-dependent CS may be challenging.

Identifying the cause of ACTH-dependent CS is essential, as it will determine the therapeutic approach. Surgical resection of the tumor is the treatment of choice in the majority of CD patients [5].

The algorithms used for differential diagnosis of CD vs. ECS are generally based on the results of biochemical and imaging tests. However, the two entities have similar biochemistry, which hinders differential diagnosis. In these cases, functional tests have higher diagnostic sensitivity and specificity.

In our patient, biochemistry was consistent with an initial diagnosis of CS, which was identified as ACTH-dependent after measuring ACTH concentrations.

On the CRH stimulation test, an increase of ≥50% in serum ACTH concentrations with respect to the baseline value confirms diagnosis of pituitary CS. The reason is that CRH receptors and signaling molecules are highly expressed in corticotrophic pituitary tumors and respond to this peptide by releasing ACTH massively, which does not occur in ectopic tumors [9]. In the case reported, the lack of response to the CRH test suggested an ectopic origin, which was inconsistent with the lack of response to suppression with 8 mg of dexamethasone, which suggested a pituitary origin.

In clinical practice, as in our case, none of the tests currently available has a diagnostic accuracy of 100%. The suppression test with high doses of dexamethasone is the gold standard non-invasive diagnostic test for CD. However, it has a sensitivity and specificity of only 60 and 80%, respectively [7, 8]. Additionally, the imaging tests used to locate the tumor may be inconclusive. Up to 40% of patients with Cushing’s disease have occult microadenomas that are not visible on pituitary MRI studies performed by experienced specialists [9]. In addition, 10% of the general population may have incidentaloma [10], [11], [12]. This is consistent with the lack of significant findings on MRI in our patient.

IPSS is the gold-standard method for differential diagnosis of CD vs. ECS. IPSS is a safe test with a high sensitivity and specificity for differential diagnosis that is also useful for the location of pituitary lesions and shows good tolerance [10]. An IPS/peripheral blood gradient (baseline (0′)) of >2 or an IPS/peripheral blood gradient (stimulated) >3 [13–15] confirms diagnosis of CD. These thresholds were applied to our case. However, these values may be influenced by the type of ACTH and cortisol assay used. In addition, the number of ECS cases included in the series for the analysis of diagnostic performance of the tests was very limited [9].

In our case, IPSS results (ACTH C/p>5 after stimulation) were essential in establishing a diagnosis of CD.

Although it is an excellent diagnostic test, IPSS has some limitations, with false negative and positive results. The most frequent cause of false-negative tests includes the presence of atrophic IPS, incorrect catheterization, or abnormal venous drainage [5]. False positives could be the result of partial suppression of the pituitary axis in cases of adrenal or ectopic Cushing’s syndrome, or in patients with cyclic or mild CS [5].

The most frequent complication of IPSS is inguinal hematoma secondary to femoral access, which occurs in less than 5% of patients. Severe complications are rare, including deep venous thrombosis, pulmonary embolism, or brain lesions [6] (hemorrhage or brain stem infarction) [15].

## Conclusions

Inferior petrosal venous sinus sampling is a safe, highly sensitive and specific diagnostic test for ACTH-dependent Cushing’s syndrome, when inconclusive results are obtained in routine complementary studies.
